# Stemness Maintenance Properties in Human Oral Stem Cells after Long-Term Passage

**DOI:** 10.1155/2017/5651287

**Published:** 2017-04-02

**Authors:** Francesca Diomede, Thangavelu Soundara Rajan, Valentina Gatta, Marco D'Aurora, Ilaria Merciaro, Marco Marchisio, Aurelio Muttini, Sergio Caputi, Placido Bramanti, Emanuela Mazzon, Oriana Trubiani

**Affiliations:** ^1^Stem Cells and Regenerative Medicine Laboratory, Department of Medical, Oral and Biotechnological Sciences, University “G. d'Annunzio”, Chieti-Pescara, Via dei Vestini 31, 66100 Chieti, Italy; ^2^IRCCS Centro Neurolesi “Bonino-Pulejo”, Via Provinciale Palermo, Contrada Casazza, 98124 Messina, Italy; ^3^Department of Psychological, Health and Territorial Sciences, School of Medicine, University “G. d'Annunzio”, Chieti-Pescara, Via dei Vestini 31, 66100 Chieti, Italy; ^4^Department of Medicine and Aging Science, University “G. d'Annunzio”, Chieti-Pescara, Via dei Vestini 31, 66100 Chieti, Italy; ^5^Department of Comparative Biomedical Sciences, University of Teramo, Via Balzarini 1, 64100 Teramo, Italy; ^6^Department of Medical, Oral and Biotechnological Sciences, University “G. d'Annunzio”, Chieti-Pescara, Via dei Vestini 31, 66100 Chieti, Italy

## Abstract

*Background*. Neural crest-derived mesenchymal stem cells (MSCs) from human oral tissues possess immunomodulatory and regenerative properties and are emerging as a potential therapeutic tool to treat diverse diseases, such as multiple sclerosis, myocardial infarction, and connective tissue damages. In addition to cell-surface antigens, dental MSCs express embryonic stem cell markers as neural crest cells originate from the ectoderm layer. In vitro passages may eventually modify these embryonic marker expressions and other stemness properties, including proliferation. In the present study, we have investigated the expression of proteins involved in cell proliferation/senescence and embryonic stem cell markers during early (passage 2) and late passages (passage 15) in MSCs obtained from human gingiva, periodontal, and dental pulp tissues. *Methods*. Cell proliferation assay, beta galactosidase staining, immunocytochemistry, and real-time PCR techniques were applied. *Results*. Cell proliferation assay showed no difference between early and late passages while senescence markers p16 and p21 were considerably increased in late passage. Embryonic stem cell markers including SKIL, MEIS1, and JARID2 were differentially modulated between P2 and P15 cells. *Discussion*. Our results suggest that the presence of embryonic and proliferation markers even in late passage may potentially endorse the application of dental-derived MSCs in stem cell therapy-based clinical trials.

## 1. Introduction

Mesenchymal stem cells (MSCs) are nonhaematopoietic stromal cells. They are self-renewable with the ability to differentiate into diverse cell types, including mesenchymal lineages (chondrocytes, fibroblasts, osteoblasts, adipocytes, and tendons) and ecto/endodermal lineages (neural cells, hepatocytes, lung cells, liver cells, pancreatic cells, cardiomyocytes, and endothelial cells [1–3]. The multipotent properties, immunomodulatory properties and the capacity to migrate into injured tissues and directly initiate tissue repair, made MSCs inevitable for regenerative medicine [4, 5]. Human MSCs can be isolated from bone marrow, adipose, dental, umbilical cord, Wharton's jelly, and placental tissues. In particular, MSCs acquired from dental tissues received greater interest due to minimal invasive practice to collect the oral tissues, autologous/allogeneic stem cell treatment options, and the ability to differentiate into various cell types in vitro [6, 7]. Six different types of human dental MSCs have been described so far: (1) dental follicle precursor cells (hDFPCs), (2) dental pulp stem cells (hDPSCs), (3) stem cells from the pulp of human exfoliated deciduous teeth (SHED), (4) stem cells from the apical papilla (hSCAP), (5) periodontal ligament stem cells (hPDLSCs), and (6) gingiva-derived stem cells (hGMSCs) [8–10]. Therapeutic role of these dental MSCs has been demonstrated in preclinical and clinical studies [11–13].

Many tissues of the craniofacial region, including the dental pulp and periodontal ligament, are originated from the ectodermal neural crest during embryogenesis [14]. Consequently, MSCs originated from these adult dental tissues express embryonic stem cell markers, such as NANOG, SSEA4, SOX2, and Oct4 [15], along with cell-surface antigens. Expression of embryonic stem cell markers in dental MSCs might be helpful in stem cell therapy-based clinical trials; however, surplus quantity of stem cells is required to accomplish any clinical trials. Thus, multiple subcultures from the initial cells are needed to obtain the optimal cell number for transplantation. Nevertheless, it is important to mention here that prolonged culture of these dental and other adult MSCs can modulate the properties of stemness, including proliferation and differentiation, over time [16–18].

In the present study, we have investigated the expression of proteins involved in cell proliferation/senescence and embryonic stem cell markers, including NANOG, SOX2, and Oct4, in hPDLSCs, hDPSCs, and hGMSCs at early passage (passage 2, P2) and late passage (passage 15, P15).

## 2. Materials and Methods

### 2.1. Ethic Statement

The study was approved by the Ethical Committee at the Medical School, “G. d'Annunzio” University, Chieti, Italy (number 266/April 17, 2014). All subjects enrolled in the study signed the informative consent form before tissue collection.

### 2.2. Cell Culture Establishment

hPDLSCs were isolated from the periodontal tissue and hDPSCs from the dental pulp of noncarious third molars extracted for orthodontic purpose, as previously described [19]. Gingival tissues were collected during surgical gingival resection for the extraction of a supernumerary tooth or for orthodontic reasons. hGMSCs were detached after their spontaneous migration from tissue samples as reported by Soundara Rajan et al. [20]. All donors were in good general health and exempt from oral and systemic diseases. Cells were cultured using MSCGM-CD medium (mesenchymal stem cell growth medium chemically defined) (Lonza, Basel, Switzerland) and were maintained in an incubator at 37°C in a humidified atmosphere of 5% CO_2_ in air. Cells were subcultured until P2 and P15. All experiments were performed in triplicate. Cells derived from each donor have been used separately.

### 2.3. Cytofluorimetric Evaluation

Samples were stained for surface or intracellular antigens (Supplementary Table 1 in Supplementary Material available online at https://doi.org/10.1155/2017/5651287), as previously described by Diomede et al. 2016 [21]. Data were analyzed using FlowJo™ software (TreeStar, Ashland, OR, USA). Mean fluorescence intensity ratio (MFI ratio) was calculated by dividing the MFI of positive events from the MFI of negative events [22].

### 2.4. Cell Viability Assay

Viability of hPDLSCs, hDPSCs, and hGMSCs at P2 and P15 were determined using the 3-(4,5-dimethylthiazolyl-2)-2,5-diphenyltetrazoliumbromide (MTT) method. 2×10^3^ cells/well were placed in a 96-well tissue culture plates and incubated at 37°C for 24, 48, 72 h, and 1 week. At each time point, MTT solution (20 *μ*l) (Promega, Milan, Italy) was added to each well to detect the metabolic activity of the cells. All plates were cultured in the dark for 3 h at 37°C. Supernatants were read at 650 nm wavelength using a microplate reader (Synergy HT, BioTek Instruments, Winooski, VT, USA). The doubling time of Trypan blue harvested cells at 24, 48, 72 h, and 1 week of culture and was calculated by using an algorithm available online (http://www.doubling-time.com).

### 2.5. Induction of Mesengenic Differentiation

Human PDLSCs, hDPSCs, and hGMSCs at P2 and P15 were induced to mesengenic differentiation as reported by Trubiani et al. [23]. Briefly, cells were stained with alizarin red S and adipo oil red solution to evaluate osteogenic and adipogenic differentiation, respectively, and observed by means of light microscopy, Leica DMIL (Leica Microsystem, Milan, Italy) [24].

### 2.6. β-Galactosidase Staining

Cells were fixed using 0.5 ml of fixative solution (supplied with Abcam Senescence detection kit (Ab65351, Abcam, Cambridge, UK)) for 10–15 min at room temperature. Subsequently, cells were stained with 0.5 ml of staining solution (20 mg/ml of X-gal) overnight at 37°C. Percentage of positively stained cells (blue cells) versus total cells was counted by randomly choosing 10 microscopic fields under ×10 objective magnification at light microscopy Leica DMIL (Leica Microsystem).

### 2.7. Immunohistochemical Analysis

hPDLSCs, hDPSCs, and hGMSCs were processed as previously reported by Trubiani et al. 2016 [23]. Antihuman *p16* (1 : 200, rabbit) (Santa Cruz Biotechnology Inc., Santa Cruz, CA, USA) and *p21* (1 : 50, rabbit) (Santa Cruz Biotechnology) were used as primary monoclonal antibodies. Subsequently, cells were incubated for 1 h at 37°C with Alexa Fluor 568 red fluorescence conjugated (1 : 200, goat antirabbit) (Molecular Probes, Invitrogen, Eugene, OR, USA), as secondary antibody.

To stain cytoskeleton actin and nuclei, cells were treated with Alexa Fluor 488 phalloidin green fluorescence conjugate (1 : 200, Molecular Probes) and TOPRO (1 : 200, Molecular Probes), respectively.

Zeiss LSM510META confocal system (Zeiss, Jena, Germany) was used to analyze stained cells, using a Plan Neofluar oil immersion objective (63×). Micrographs were obtained using excitation lines at 488 nm for argon laser beam and at 543 and 665 nm for a helium-neon source.

### 2.8. Western Blot Analysis

Western blot procedure was performed as previously described by Rajan et al. 2016 [25]. *p16* (Bethyl Laboratories Inc., Montgomery, TX, USA; 1 : 4000) and *p21* (Santa Cruz Biotechnology, Santa Cruz, CA, USA; 1 : 500) were used as primary antibodies. *β*-Actin (Santa Cruz Biotechnology; 1 : 750) was used to assess the uniform protein loading. Bands were analyzed by the ECL method using Alliance 2.7 (UVItec Limited, Cambridge, UK).

### 2.9. RNA Extraction and TaqMan Quantitative Real-Time PCR

Total RNA was extracted from hPDLSCs, hDPSCs, and hGMSCs at P2 and P15 using the RNeasy Mini Kit (Quiagen, Hilden, Germany). 2 *μ*g of RNA from each sample was reverse transcribed using the High Capacity RNA-to-cDNA Kit (Applied Biosystems, Foster, UK). To analyze mesengenic differentiation, RUNX-2, ALP, PPAR*γ*, and FABP4 markers were evaluated as reported by Cianci et al. [26]. Moreover, quantitative real-time PCR was performed on a 96-well TaqMan® Array Human Transcriptional Regulatory Network in Embryonic Stem Cell following manufacturer's instructions and run on an Abi 7900HT Sequencing Detection System (Applied Biosystems). Each plate contains 42 assays related to stem cells pluripotency including a battery of transcription factors such as Oct4 (octamer-binding transcription factor-4), SOX2 (SRY (sex-determining region Y) box-2), and NANOG (Nanog homeobox) and 4 housekeeping genes. Among transcriptionally inactive genes co-occupied by Oct4, SOX2, and NANOG, genes that specify transcription factors important for differentiation into extraembryonic, endodermal, mesodermal, and ectodermal lineages (e.g., ESX1L (Extraembryonic, Spermatogenesis, Homeobox-1 Homolog (Mouse)), HOXB1 (Homeobox-B1), HAND1 (Heart And Neural Crest Derivatives Expressed-1), MEIS1 (Meis Homeobox-1), PAX6 (Paired Box-6), LHX5 (LIM Homeobox-5), MYF5 (Myogenic Factor-5), and ONECUT1 (One Cut Homeobox-1)) are present. The amplification cycle was 10 minutes at 95°C followed by 40 cycles of 15 seconds at 95°C and 1 minute at 60°C. Three independent experiments were run for each condition for a total of 18 plates. Each sample was run as a duplicate in the same plate. Real-time data were analyzed by DataAssist software (Applied Biosystems). A global normalization analysis was used, and GAPDH, 18s, and HPRT1 were chosen as selected internal controls. Only genes showing no outlier replicates and a maximum Ct value = 35 were included in the analysis. A gene was considered differentially expressed when showing a *p* value < 0.05; *p* values were adjusted using Benjamini-Hochberg FDR correction. Ingenuity Pathway Analysis (IPA) software (Ingenuity Systems, Redwood City, CA, USA) was employed to infer biological functions of the selected gene datasets. IPA predicts functional characterization based on known gene functional interactions and ranks them by a significance score [27].

*p16* and *p21* gene expressions were analyzed by qRT-PCR using the same cDNA employed for expression arrays. Specific primer and probe sets employed were purchased from ThermoFisher Scientific (Waltham, MA, USA): *p16* Hs00923894_m1 and *p21* Hs01040810_m1. qRT-PCR was performed in a total volume of 30 *μ*l containing KAPA Probe Fast Abi Prism qPCR Kit (KAPA Biosystems), 25 ng of cDNA, and 1 *μ*l of primer-probe mixture (20x) on an Abi 7900HT Sequencing Detection System. The selected gene relative expression was corrected against GAPDH (Hs02758991) used as endogenous control (ThermoFisher Scientific, Waltham, MA, USA). Real-time amplification conditions were as follows: 10 minutes at 95°C followed by 40 cycles of 15 seconds at 95°C, and 1 minute at 60°C. Each sample was run as triplicate. The ΔΔCt method was used to compare relative fold changes between samples and control. *t*-test was used to assess the *p* value, considering data significant when *p* < 0.05.

### 2.10. Statistical Analysis

Data were analyzed using GraphPad Prism 6.0 (GraphPad Software, La Jolla, CA, USA) with one-way ANOVA test, followed by a Bonferroni post hoc test for multiple comparisons. A *p* value < 0.05 was considered statistically significant.

## 3. Results

### 3.1. Cytofluorimetric Evaluation

The short- and long-term passages hPDLSCs, hDPSCs, and hGMSCs were characterized for the expression of stem cell markers. In particular, they showed a positivity for CD13, CD29, CD44, CD73, CD90, CD105, CD166, HLA-ABC, NANOG, OCT4, SSEA4, and SOX2. On the contrary, all cells were negative for the following markers: CD14, CD31, CD34, CD45, CD117, CD133, CD326, and HLA-DR (Tables 1, 2, and 3).

### 3.2. Proliferation Analysis

**Figure 1:**
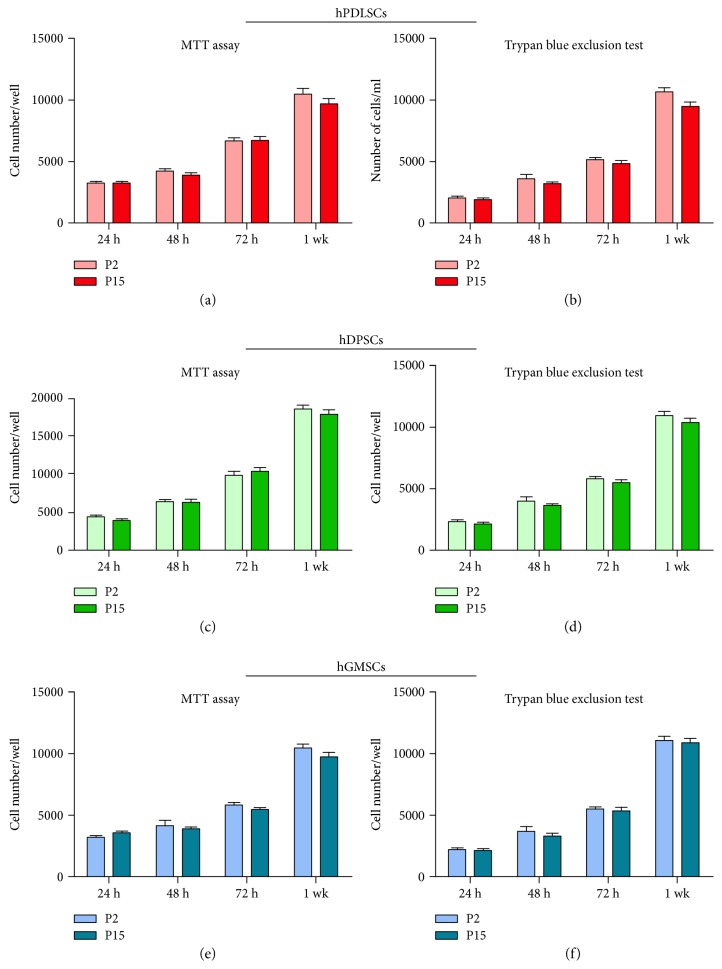
Cell viability and proliferation. Graphs show the proliferation rate at different time of each cell primary cultures at P2 and P15. Bar graphs display the exponential growth pattern of (a) hPDLSCs, (c) hDPSCs, and (e) hGMSCs, evaluated by MTT assay. Proliferation rate of (b) hPDLSCs, (d) hDPSCs, and (f) hGMSCs, performed by Trypan blue exclusion test, confirmed MTT assay results. Cells showed a logarithmic proliferation trend at P2 and P15 without any statistically significant differences. The *y*-axis shows cell number and *x*-axis shows culture time. Results in each bar graph are the composite data from experiments performed in triplicate (mean ± SEM).

hPDLSC, hDPSC, and hGMSC proliferations were measured with commercially available MTT proliferation assay kit (Figure 1). The proliferative rate was detected at 24, 48, 72 h, and 1 week of culture. The difference among short and long passage-cultured cells was not statistically significant among hPDLSCs, hDPSCs, and hGMSCs (Figures 1(a), 1(c), and 1(e), resp.).

MTT data were confirmed by Trypan blue exclusion test staining. The results from Trypan blue staining of hPDLSCs, hDPSCs, and hGMSCs at P2 and P15 displayed the logarithmic growth during the culture time (Figures 1(b), 1(d), and 1(f), resp.).

### 3.3. Mesengenic Differentiation

**Figure 2:**
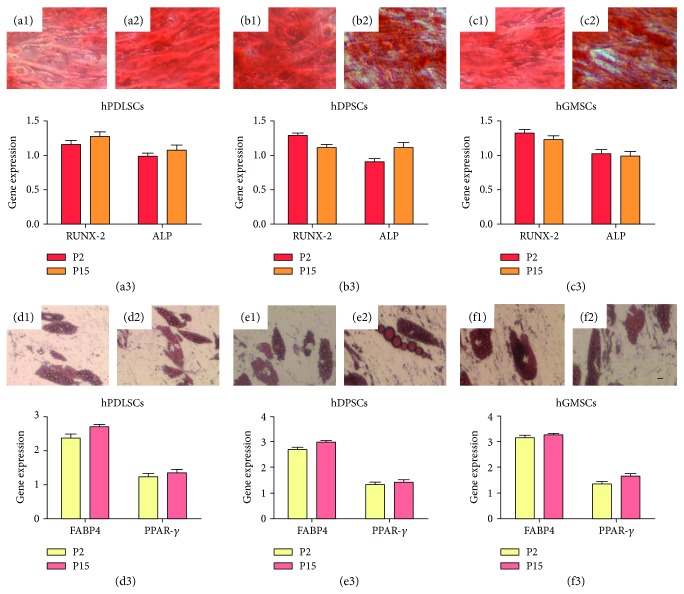
Mesengenic differentiation potential. hPDLSCs, hDPSCs, and hGMSCs induced to osteogenic commitment, stained with alizarin red S solution at P2 (a1, b1, and c1) and P15 (a2, b2, and c2) showed no statistical significative differences among two different culture stages. RUNX-2 and ALP expressions confirm light microscopy observations for osteogenic commitment (a3, b3, and c3). Adipogenic induction analyzed by oil red solution staining demonstrates the presence of lipid vacuoles at cytoplasmic level in cells cultured at early (d1, e1, and f1) and late passages (d2, e2, and f2). Both PPAR*γ* and FABP4 showed no statistical differences after 28 days of culture, under differentiation conditions, at P2 and P15 (d3, e3, and f3). Mag.: 10x, bars: 10 *μ*m.

To evaluate osteogenic differentiation, cells at P2 and P15 were stained with alizarin red S solution. Calcium precipitates were detected at same levels in both P2 and P15 of all hPDLSCs, hPDSCs, and hGMSCs (Figures 2(a1), 2(a2), 2(b1), 2(b2), 2(c1), and 2(c2)). To confirm that cells maintain differentiation ability at P2 and P15, RUNX-2 and ALP were analyzed by qRT-PCR (Figures 2(a3), 2(b3), and 2(c3), resp.). In addition, to evaluate differentiation to adipogenic lineage, cells were stained with oil red O solution to highlight lipid droplet accumulation at cytoplasmic level (Figures 2(d1), 2(d2), 2(e1), 2(e2), 2(f1), and 2(f2)). PPAR*γ* and FABP4, adipogenic-related markers, were expressed with no significant differences among P2 and P15 cells (Figures 2(d3), 2(e3), and 2(f3)). Both mesengenic differentiations showed no statistically significant differences between groups.

### 3.4. Senescence Marker Evaluation

**Figure 3:**
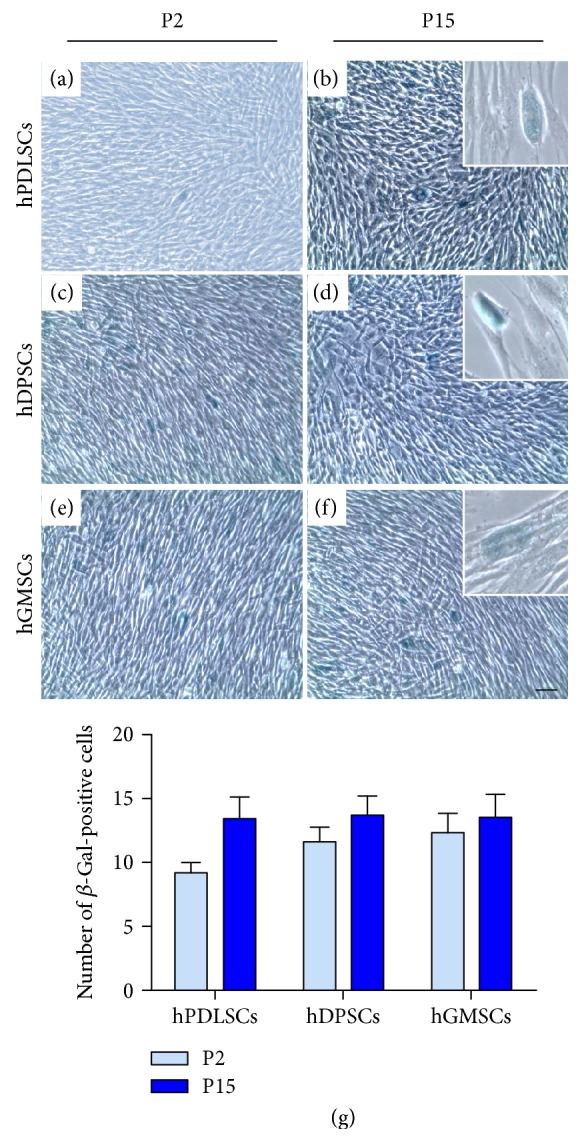
Senescence-associated *β*-galactosidase staining. (a, c, and e) hPDLSCs, hDPSCs, and hGMSCs from P2 were stained with X-gal solution. (b, d, and f) hPDLSCs, hDPSCs, and hGMSCs at P15 were stained with X-gal solution and showed low levels of blue precipitation (Mag.: 10x). (b, d, and f) X-gal positive staining of hPDLSCs, hDPSCs, and hGMSCs at high magnification (Mag.: 40x). (g) Percentage of *β*-gal-positive cells. Results are representative of three independent experiments. Bar: 10 *μ*m.

We checked hPDLSCs, hDPSCs, and hGMSCs at P2 and P15 for senescent marker *β*-galactosidase. At P2, few numbers of positive X-gal solution-stained cells were detectable (Figures 3(a), 3(c), and 3(e)). Interestingly, the *β*-galactosidase staining showed slightly more positivity only in hPDLSCs, hDPSCs, and hGMSCs at P15 (Figures 3(b), 3(d), and 3(f), resp.). High magnification pictures put in evidence the specific positive granular staining at cytoplasmic levels (Figures 3(b), 3(d), and 3(f)).  Figure 3(g) showed the percentage of *β*-gal-positive cells evaluated at P2 and P15, validating the results obtained at light microscopy.

Labelling of the *p16* senescence marker showed a slight positivity at P15 for hPDLSCs (Figure S1B2) and hDPSCs (Figure S2B2), while basal staining was noticed at P2 for hPDLSCs (Figure S1A2) and hDPSCs (Figure S2A2). Minimal staining of *p16* was observed at both P2 and P15 in hGMSCs (Figures S3A2 and S3B2, resp.). Another senescence marker, *p21*, showed negligible staining at P2 and a low positive staining at P15 in hPDLSCs (Figures S1C2 and S1D2, resp.). Same results were obtained at P2 and P15 of hDPSCs (Figures S2C2 and S2D2, resp.) and hGMSCs (Figures S3C2 and S3D2, resp.).

### 3.5. p16 and p21 Analysis

**Figure 4:**
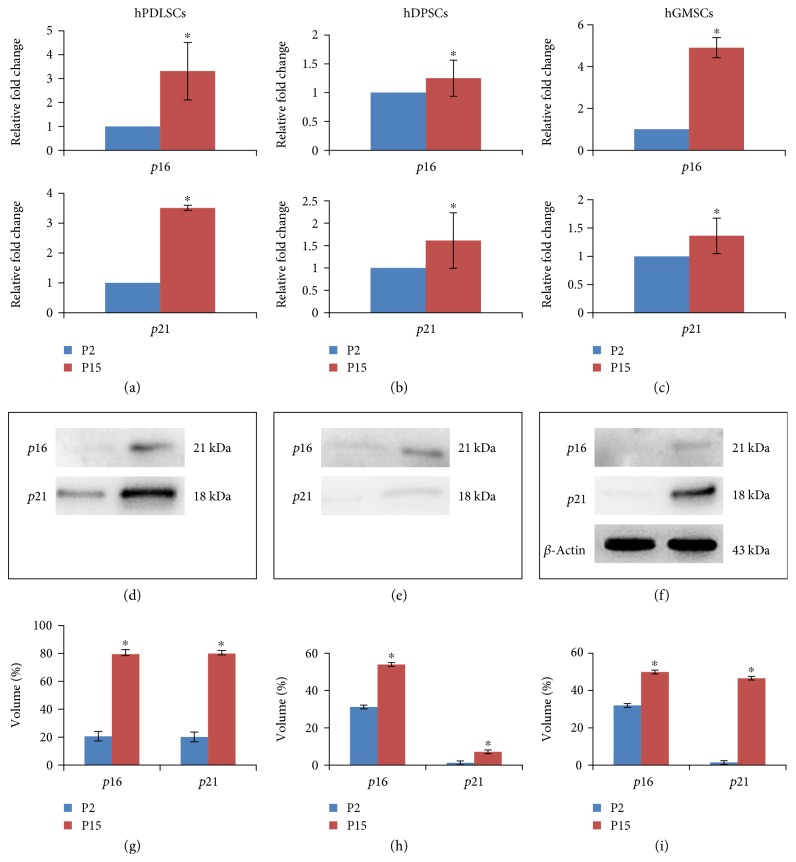
Characterization of early and late passages. (a) qRT-PCR for *p16* and *p21*. The relative fold changes represent differences in gene expression at P15 compared to P2 in hPDLSCs. *p16* and *p21* gene expressions showed a statistically significant difference at passage 15 (*p* < 0.05). (d) *p16* and *p21* protein expressions at P2 and P15 hPDLSCs were validated by means of Western blotting analysis. (b) The relative fold changes represent differences in gene expression at P15 compared to P2 in hDPSCs. *p16* and *p21* gene expressions showed a statistically significant difference at passage 15 when compared to P2 (*p* < 0.05). (e) Western blotting analysis of *p16* and *p21* validated gene expression profile at P2 and P15 of hDPSCs. (c) The relative fold changes represent differences in gene expression at P15 compared to P2 in hGMSCs. *p16* and *p21* gene expressions showed a statistical significance at passage 15 (*p* < 0.05). (f) Western blotting analysis of *p16* and *p21* at P2 and P15 hGMSCs confirmed gene expression analyses. (g, h, and i) Densitometric analysis of bands related to *p16* and *p21* at P2 and P15 in hPDLSCs, hDPSCs, and hGMSCs (*p* < 0.05). Statistical analyses were performed using one-way ANOVA and Bonferroni post hoc test.

*p16* and *p21* showed a slightly increased expression pattern at P15 when compared to P2 in all three primary cultures (*p* < 0.05) (Figures 4(a), 4(b), and 4(c)). According to RT-PCR results, western blot showed an upregulation of both senescence markers examined in hPDLSCs, hDPSCs, and hGMSCs at P15. In particular, a low expression of *p16* and *p21* was noticed at P2 in all primary cultures (Figures 4(d), 4(e), and 4(f)). Densitometric analysis of specific bands confirmed *p16* and *p21* expressions in hPDLSCs, hDPSCs, and hGMSCs (*p* < 0.05) (Figures 4(g), 4(h), and 4(i), resp.). *β*-Actin was used as a loading control protein.

### 3.6. Gene Expression

**Figure 5:**
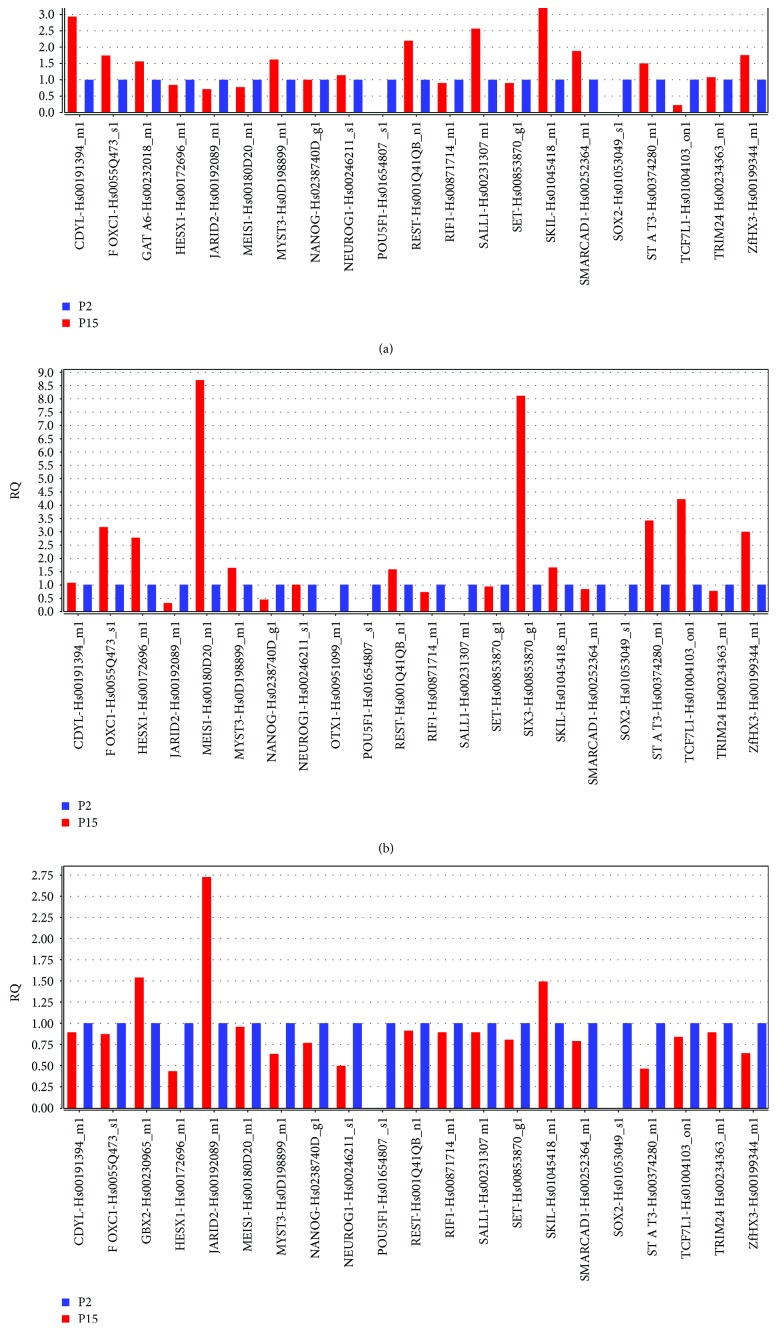
Relative gene expression folds by qRT-PCR. Bar charts show the significant relative gene expression folds in (a) hGMSCs, (b) hPDLSCs, (c) and hDPSCs at P15 under culturing conditions compared to P2. Expression levels of transcripts at P2 and P15 are shown in blue and red, respectively. DataAssist software was employed to run a global normalization analysis by using GAPDH, 18s, and HPRT1 as selected internal controls. The reported transcripts evidenced a *p* value < 0.05; *p* values were adjusted using Benjamini-Hochberg FDR correction.

**Figure 6:**
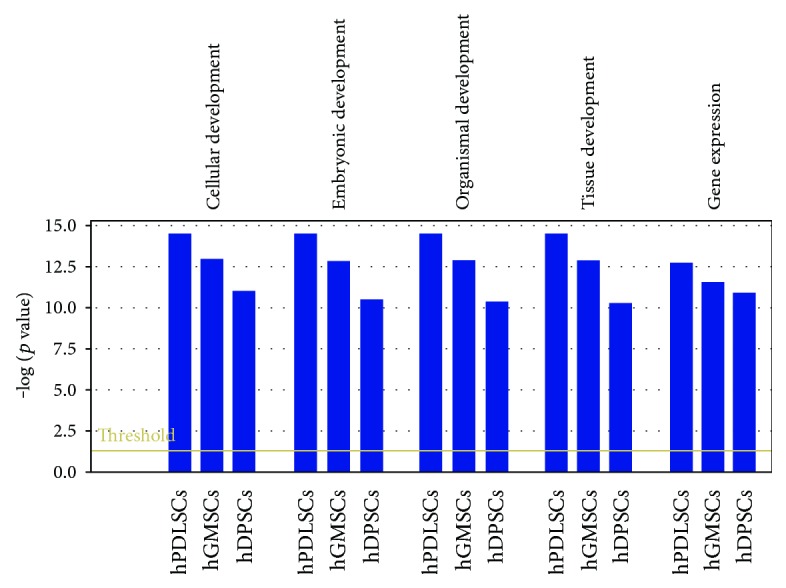
IPA functional analysis of the three gene datasets. IPA biological function analysis shows key functions modulated at P15 by the selected genes for hPDLSCs, hGMSCs, and hDPSCs.

In order to validate the capability of the stem cell lines and to maintain proliferation and differentiation abilities after prolonged in vitro cultures, we analyzed the gene expression signature of transcriptional regulatory network in embryonic stem cell in hGMSCs, hPDLSCs, and hDPSCs at P15 under culture conditions, compared to P2. Human PDLSCs significantly expressed 22 genes, hDPSCs displayed significant modulation of 21 genes, and hGMSCs showed significant expression of 21 genes in the pathway at passage 15 (Figures 5(a), 5(b), and 5(c)). Moreover, all cells at P15 showed a strong reduction in the expression of POU5F1 and SOX2 while NANOG was downregulated of about 50% in hPDLSCs and hDPSCs; however, NANOG expression was unchanged in hGMSCs. The pathway gene expression modulation is similar for hGMSCs and hPDLSCs displaying the overexpression of transcripts such as FOXC1, MEIS1, STAT3, and ZFHX3 while hDPSCs reveal a unique gene signature with the overexpression of only three transcripts (GBX2, JARID2, and SKIL). IPA functional analysis of the three gene datasets revealed that these genes are mainly involved in cellular, embryonic, and tissue development and in gene expression regulation (Figure 6). IPA analysis revealed that hGMSCs and hPDLSCs have an increase in differentiation process, in particular ectodermal differentiation whereas hDPSCs showed a reduction in transcriptional activity and a lower differentiation pattern, although overexpressing JARID2 and SKIL which are involved in osteo- and muscular-differentiation patterns (data not shown).

**Figure 7:**
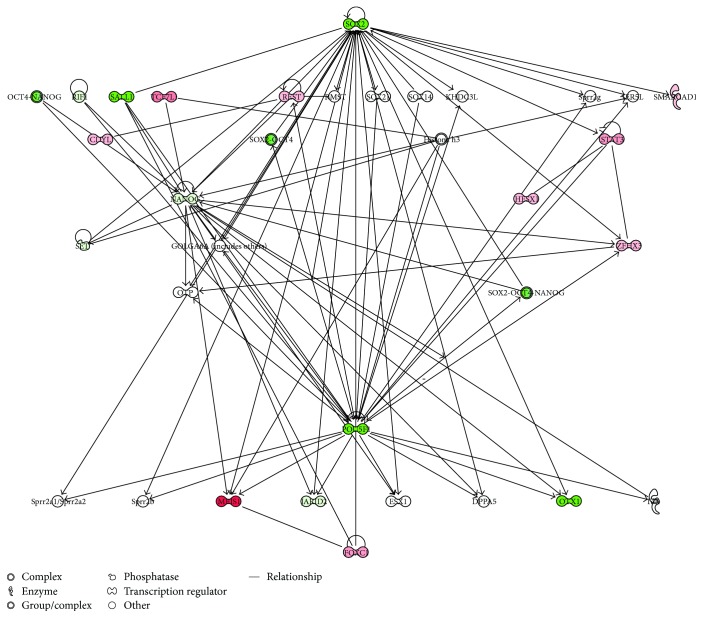
Gene modulation of hDPLSCs. IPA-inferred cluster analysis of the hPDLSC dataset of significantly expressed genes at P15.

**Figure 8:**
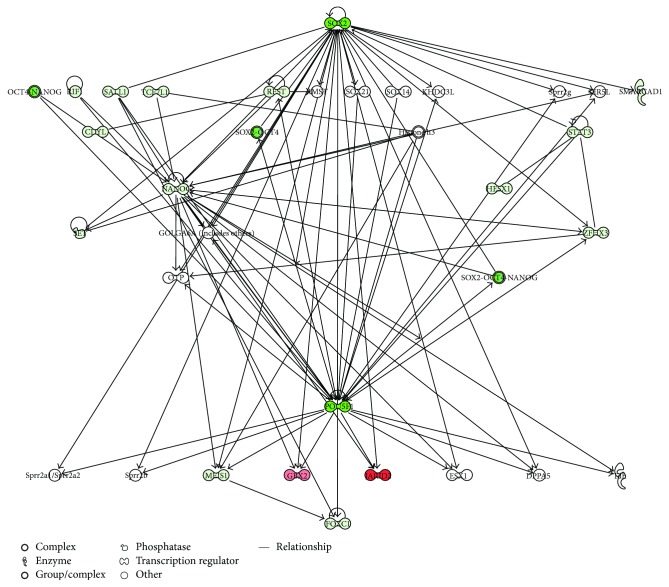
Gene modulation of hDPSCs. IPA-inferred cluster analysis of the hDPSC dataset of significantly expressed genes at P15.

**Figure 9:**
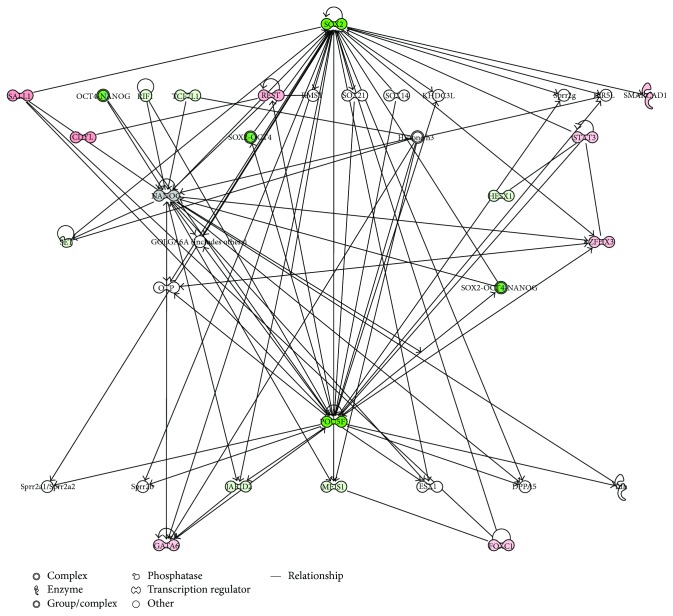
Gene modulation of hGMSCs. IPA-inferred cluster analysis of the hGMSC dataset of significantly expressed genes at P15.

IPA-inferred gene network analysis of the three gene datasets demonstrated that at P15 the downregulation of SOX2 and POU5F1 triggers the modulation of the analyzed transcripts in a different manner for the three conditions (Figures 7, 8, and 9). In fact, the intracellular pathways were differentially regulated, showing the specific activation of HESX1, MEIS1, and TCF7L1 in hPDLSCs; the activation of CDYL, GATA6, SALL1, and SMARCAD1 in hGMSCs; and the activation of GBX2, JARID2, and SKIL in hDPSCs. IPA functional analysis demonstrated that this differential gene expression modulation is due to the ability of these cells to differentiate in specific lineages.

## 4. Discussion

Research in recent years attested that dental tissues serve as an alternative source for MSCs and regenerative application of dental MSCs has been demonstrated not only in dental regeneration but also in other diseases [28]. Together with stem cell-associated cell surface markers, owing to their neural crest origin, dental MSCs express embryonic stemness markers as well [29], which may influence them to differentiate into diverse cell lineages in vitro.

MSCs from oral tissues can be considered an interesting accessible autologous platform of stem cells, providing an alternative source for regenerative medicine. In particular, dental pulp and periodontal ligament express proteins similar to BMSCs and can be induced toward osteogenic, adipogenic, chondrogenic, and neurogenic differentiation [26, 30].

Large-scale expansion of MSCs with low grade of senescence is very crucial for stem cell transplantation. However, continuous passages of adult MSCs for a longer period may affect the embryonic stemness properties, including proliferation and differentiation markers [17, 18].

In the present study, we have investigated the expression of embryonic markers and proteins involved in proliferation/senescence in hPDLSCs, hDPSCs, and hGMSCs at two different passages: P2 and P15. All oral primary cultures express surface antigen markers linked with MSCs, such as CD13, CD29, CD44, CD73, CD90, CD105, CD166, HLA-ABC, NANOG, OCT4, SSEA4, and SOX2. In addition, we noticed that the degree of cell proliferation at P2 and P15 remain unchanged among hPDLSCs, hDPSCs, and hGMSCs. These results suggest that these dental MSCs are highly proliferative even at P15. Cell proliferation efficacy of hPDLSCs, hDPSCs, and dental periapical follicle MSCs has been studied previously where it has been reported that hDPSCs showed adequate proliferation between passages 11–14 [31] and that periapical follicle MSCs displayed greater cell proliferation than hDPSCs and hPDLSCs [32]. However, in our study, we did not find any difference in the cell proliferation rate in hPDLSCs, hDPSCs, and hGMSCs at both P2 and P15.

We then analyzed the expression of senescence markers in hPDLSCs, hDPSCs, and hGMSCs. Continuous passages may likely induce senescence in MSCs and that cellular senescence contributes to aging and age-related diseases [33], which possess danger to transplant long-term-cultured MSCs for stem cell therapy. Senescence-associated *β*-galactosidase staining showed less positivity at only P15, while no staining was noticed at P2 in all the dental MSCs. On the other hand, senescence marker *p16* expression was slightly increased at the 15th passage in hPDLSCs and hDPSCs and not in hGMSCs. Meanwhile, *p21*, another senescence marker, remained unchanged at both P2 and P15 in hPDLSCs, hDPSCs, and hGMSCs. Additional experiments with more numbers of passages need to be performed to study senescence phenomena in these dental MSCs.

Lastly, we studied the expression of selective embryonic stemness markers at early and late passages. RT-PCR technique revealed that embryonic markers OCT4, SSEA4, SOX2, and NANOG were present at P2 of hPDLSCs, hDPSCs, and hGMSCs. Other embryonic markers SALL1 and OTX1 were absent in P15 of hPDLSCs. NANOG expression was diminished in P15 of hPDLSCs and hDPSCs, while it remained unaltered in hGMSCs. In addition, we noticed heterogeneous expression of other embryonic markers such as CDYL, FOXC1, HESX1, JARID2, MEIS1, and MYST3 at P15 in hPDLSCs, hDPSCs, and hGMSCs. A varied expression of embryonic stemness markers in MSCs obtained from the human bone marrow, adipose tissue, heart, and dermis at early passages has been already reported previously [34]. Although derived from neural crest tissues, results from our data demonstrated that MSCs of different dental tissues might acquire different changes in markers associated with the stemness properties over prolonged culture expansion.

Neurogenic markers such as NEUROG1, REST, and ZFHX3 were differentially modulated among hPDLSCs, hDPSCs, and hGMSCs at P15, which assume that continuous subculture of these dental MSCs may preferably channel them to differentiate into neural progenitor cells. Considering the neural crest origin of dental MSCs, additional experiments must be performed to investigate the potential of dental tissue-derived MSCs on autonomous neurogenesis differentiation. Expression of markers involved in the regulation of cell growth, proliferation, and DNA repair mechanism such as RIF1 and SET was downregulated, while SKIL expression was upregulated at P15 in hPDLSCs, hDPSCs, and hGMSCs. Other cell proliferation markers SMARCAD1, TCFL1, and TRIM24 were differentially modulated at P15. These results corroborate our cell proliferation data, suggesting marked rate of proliferation at P15 in hPDLSCs, hDPSCs, and hGMSCs.

## 5. Conclusion

In summary, our results demonstrated that hPDLSCs, hDPSCs, and hGMSCs do proliferate at a similar rate in P2 and P15. Senescence marker *p16* was only slightly modified in the late passage, while *p21* remained unchanged. Embryonic stemness markers were differentially modulated in P15. We conclude that expression of embryonic and proliferation markers at late passage with mild regulation of senescence may potentially recommend the use of oral-derived MSCs in stem cell therapy.

## Conflicts of Interest

The authors declare that there is no conflict of interest regarding the publication of this paper.

## Supplementary Material

Table 1: List of antibodies and antibodies suppliers.
Figure S1: Immunocytochemistry analysis of senescence markers p16 and p21 in hPDLSCs.
Figure S2: Immunocytochemistry analysis of p16 and p21 in hDPSCs.
Figure S3: Immunocytochemistry analysis of p16 and p21 in hGMSCs.

## Figures and Tables

**Table 1:** Cytofluorimetric analysis of hPDLSCs.

Flow cytometry phenotyping of hPDLSCs				
Antigen	Phenotype	hPDLSC MFI ratio ± SD		
		P2	P15	*p* value
CD13	+++	172.4 ± 38.1	157.6 ± 42.5	0.456
CD14	−	1.1 ± 0.2	1.2 ± 0.2	0.104
CD29	+++	198.6 ± 53.1	143.6 ± 43.2	0.099
CD31	−	1.3 ± 0.3	1.2 ± 0.1	0.462
CD34	−	1.2 ± 0.2	1.1 ± 0.1	0.306
CD44	+++	166.7 ± 32.6	124.1 ± 33.9	0.107
CD45	−	1.3 ± 0.1	1.1 ± 0.1	0.193
CD73	+	29.2 ± 8.9	22.3 ± 5.9	0.169
CD90	+++	396.1 ± 42.4	216.3 ± 37.3	**↓0.016**
CD105	+	9.1 ± 3.4	5.5 ± 1.3	0.134
CD117	−	1.1 ± 0.2	1.2 ± 0.2	0.349
CD133	−	1.2 ± 0.3	1.3 ± 0.3	0.347
CD166	+	18.7 ± 8.5	20.7 ± 8.2	0.439
CD326	−	1.3 ± 0.2	1.4 ± 0.1	0.310
HLA-ABC	++	127.1 ± 48.1	93.9 ± 21.1	0.283
HLA-DR	−	1.2 ± 0.1	1.4 ± 0.1	0.396
NANOG	+	7.7 ± 2.2	8.6 ± 1.7	0.127
OCT4	+	3.1 ± 0.4	3.1 ± 0.5	0.429
SSEA4	+	4.7 ± 1.6	4.9 ± 1.2	0.204
SOX2	+	60.1 ± 23.3	66.4 ± 18.4	0.394

− indicates negative expression (0%); + indicates moderate expression; ++ indicates positive; +++ indicates high expression (100%); MFI ratio is the average of five different biological samples ± standard deviation; bold values represent MFI ratio with *p* ≤ 0.05; cutoff MFI ratio positivity > 2.

**Table 2:** Cytofluorimetric analysis of hDPSCs.

Flow cytometry phenotyping of hDPSCs				
Antigen	Phenotype	hDPSC MFI ratio ± SD		
		P2	P15	*p* value
CD13	++	140.3 ± 37.7	145.1 ± 15.9	0.425
CD14	−	1.2 ± 0.2	1.3 ± 0.2	0.260
CD29	+++	235.7 ± 24.8	150.2 ± 27.5	**↓0.022**
CD31	−	1.5 ± 0.3	1.7 ± 0.1	0.153
CD34	−	1.2 ± 0.2	1.4 ± 0.1	0.208
CD44	++	92.1 ± 21.1	99.8 ± 24.3	0.446
CD45	−	1.2 ± 0.1	1.1 ± 0.1	0.463
CD73	++	58.3 ± 8.9	44.9 ± 6.6	0.075
CD90	+++	221.7 ± 39.7	411.5 ± 36.7	**↑0.007**
CD105	+	8.4 ± 3.1	6.2 ± 1.9	0.204
CD117	−	1.3 ± 0.2	1.4 ± 0.2	0.198
CD133	−	1.7 ± 0.3	1.3 ± 0.3	0.401
CD166	+	11.1 ± 4.8	17.9 ± 3.3	0.055
CD326	−	1.1 ± 0.2	1.1 ± 0.1	0.287
HLA-ABC	++	84.7 ± 6.8	79.6 ± 8.2	0.246
HLA-DR	−	1.2 ± 0.1	1.4 ± 0.1	0.399
NANOG	+	5.9 ± 2.5	6.9 ± 2.4	0.261
OCT4	+	2.5 ± 0.3	2.5 ± 0.4	0.171
SSEA4	+	4.6 ± 0.8	4.1 ± 0.6	0.377
SOX2	+	44.9 ± 6.8	45.1 ± 8.8	0.119

− indicates negative expression (0%); + indicates moderate expression; ++ indicates positive; +++ indicates high expression (100%); MFI ratio is the average of five different biological samples ± standard deviation; bold values represent MFI ratio with *p* ≤ 0.05; cutoff MFI ratio positivity > 2.

**Table 3:** Cytofluorimetric analysis of hGMSCs.

Flow cytometry phenotyping of hGMSCs				
Antigen	Phenotype	hGMSC MFI ratio ± SD		
		P2	P15	*p* value
CD13	+++	150.7 ± 40.4	299.7 ± 48.9	0.057
CD14	−	1.1 ± 0.2	1.3 ± 0.2	0.079
CD29	++	113.1 ± 20.2	106.8 ± 18.7	0.356
CD31	−	1.2 ± 0.3	1.4 ± 0.1	0.139
CD34	−	1.1 ± 0.2	1.2 ± 0.1	0.437
CD44	+++	163.9 ± 31.5	143.4 ± 33.9	0.245
CD45	−	1.2 ± 0.1	1.1 ± 0.1	0.230
CD73	+	29.2 ± 8.9	22.3 ± 5.9	0.171
CD90	+++	273.1 ± 58.9	156.7 ± 36.7	0.092
CD105	+	9.4 ± 1.7	5.8 ± 1.2	0.085
CD117	−	1.2 ± 0.2	1.3 ± 0.2	0.211
CD133	−	1.4 ± 0.3	1.2 ± 0.3	0.253
CD166	+	16.5 ± 2.5	20.9 ± 2.7	0.090
CD326	−	1.2 ± 0.2	1.1 ± 0.1	0.277
HLA-ABC	++	112.1 ± 35.4	86.6 ± 39.1	0.398
HLA-DR	−	1.3 ± 0.1	1.2 ± 0.1	0.403
NANOG	+	7.8 ± 2.5	6.0 ± 1.9	0.225
OCT4	+	4.3 ± 0.7	1.7 ± 0.5	**↓0.019**
SSEA4	+	3.4 ± 0.8	5.4 ± 1.4	0.148
SOX2	+	44.9 ± 6.8	45.1 ± 8.8	0.193

− indicates negative expression (0%); + indicates moderate expression; ++ indicates positive; +++ indicates high expression (100%); MFI ratio is the average of five different biological samples ± standard deviation; bold values represent MFI ratio with *p* ≤ 0.05; cutoff MFI ratio positivity > 2.
