# Supplementary material for: Stemness Maintenance Properties in Human Oral Stem Cells after Long-Term Passage
Source: Stem Cells Int. 2017 Apr 2;2017:5651287. doi: 10.1155/2017/5651287 (PMC5392399; doi:10.1155/2017/5651287)
Supplement: Supplementary file 1 — Table 1: List of antibodies and antibodies suppliers. Figure S1: Immunocytochemistry analysis of senescence markers p16 and p21 in hPDLSCs. Figure S2: Immunocytochemistry analysis of p16 and p21 in hDPSCs. Figure S3: Immunocytochemistry analysis of p16 and p21 in hGMSCs. [file 5651287.f1.pptx]

## Slide 1
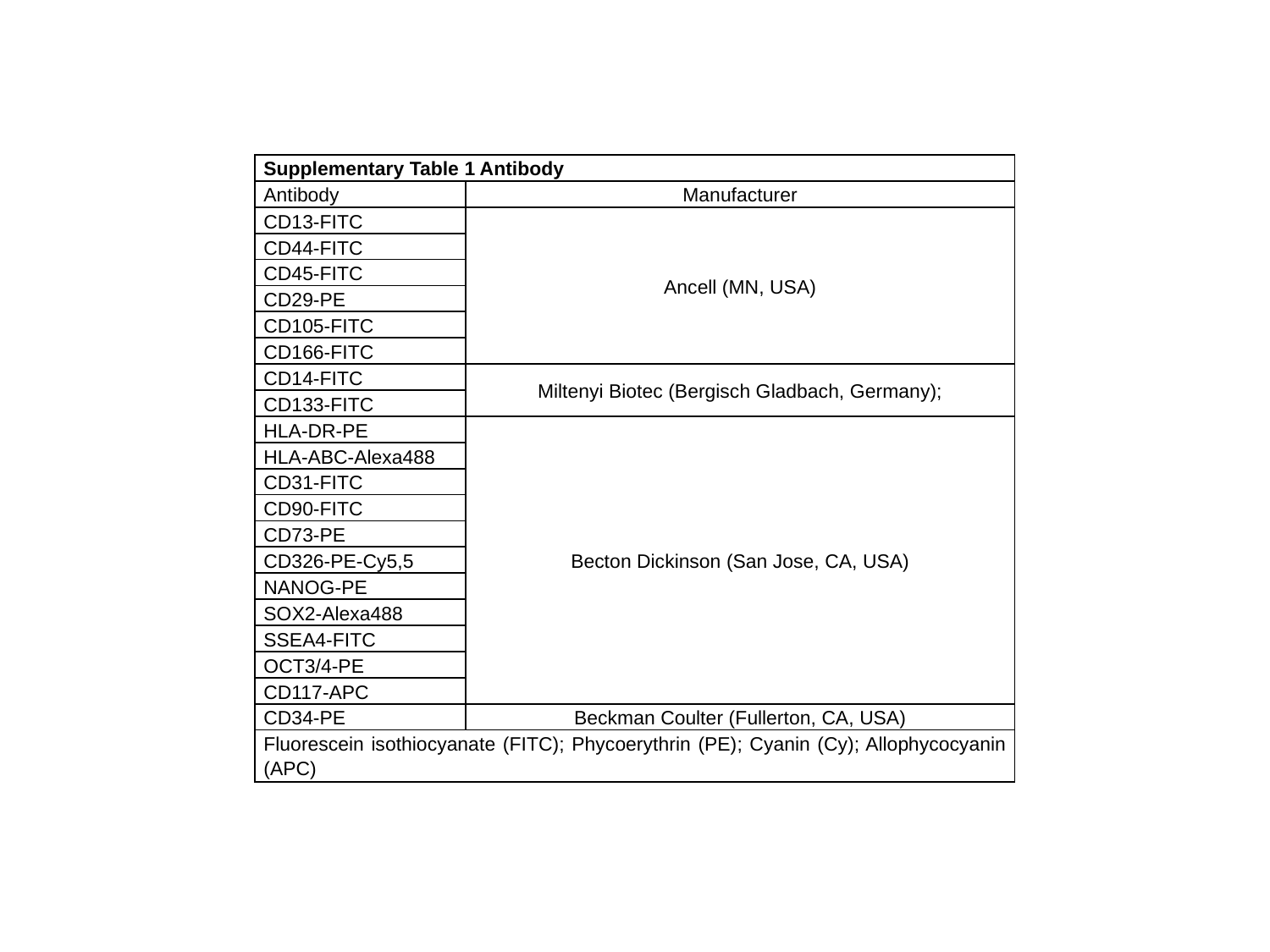

| Supplementary Table 1 Antibody | |
| --- | --- |
| Antibody | Manufacturer |
| CD13-FITC | Ancell (MN, USA) |
| CD44-FITC | |
| CD45-FITC | |
| CD29-PE | |
| CD105-FITC | |
| CD166-FITC | |
| CD14-FITC | Miltenyi Biotec (Bergisch Gladbach, Germany); |
| CD133-FITC | |
| HLA-DR-PE | Becton Dickinson (San Jose, CA, USA) |
| HLA-ABC-Alexa488 | |
| CD31-FITC | |
| CD90-FITC | |
| CD73-PE | |
| CD326-PE-Cy5,5 | |
| NANOG-PE | |
| SOX2-Alexa488 | |
| SSEA4-FITC | |
| OCT3/4-PE | |
| CD117-APC | |
| CD34-PE | Beckman Coulter (Fullerton, CA, USA) |
| Fluorescein isothiocyanate (FITC); Phycoerythrin (PE); Cyanin (Cy); Allophycocyanin (APC) | |
