# Supplementary material for: Stemness Maintenance Properties in Human Oral Stem Cells after Long-Term Passage
Source: Stem Cells Int. 2017 Apr 2;2017:5651287. doi: 10.1155/2017/5651287 (PMC5392399; doi:10.1155/2017/5651287)
Supplement: Supplementary file 2 [file 5651287.f2.pptx]

## Slide 1
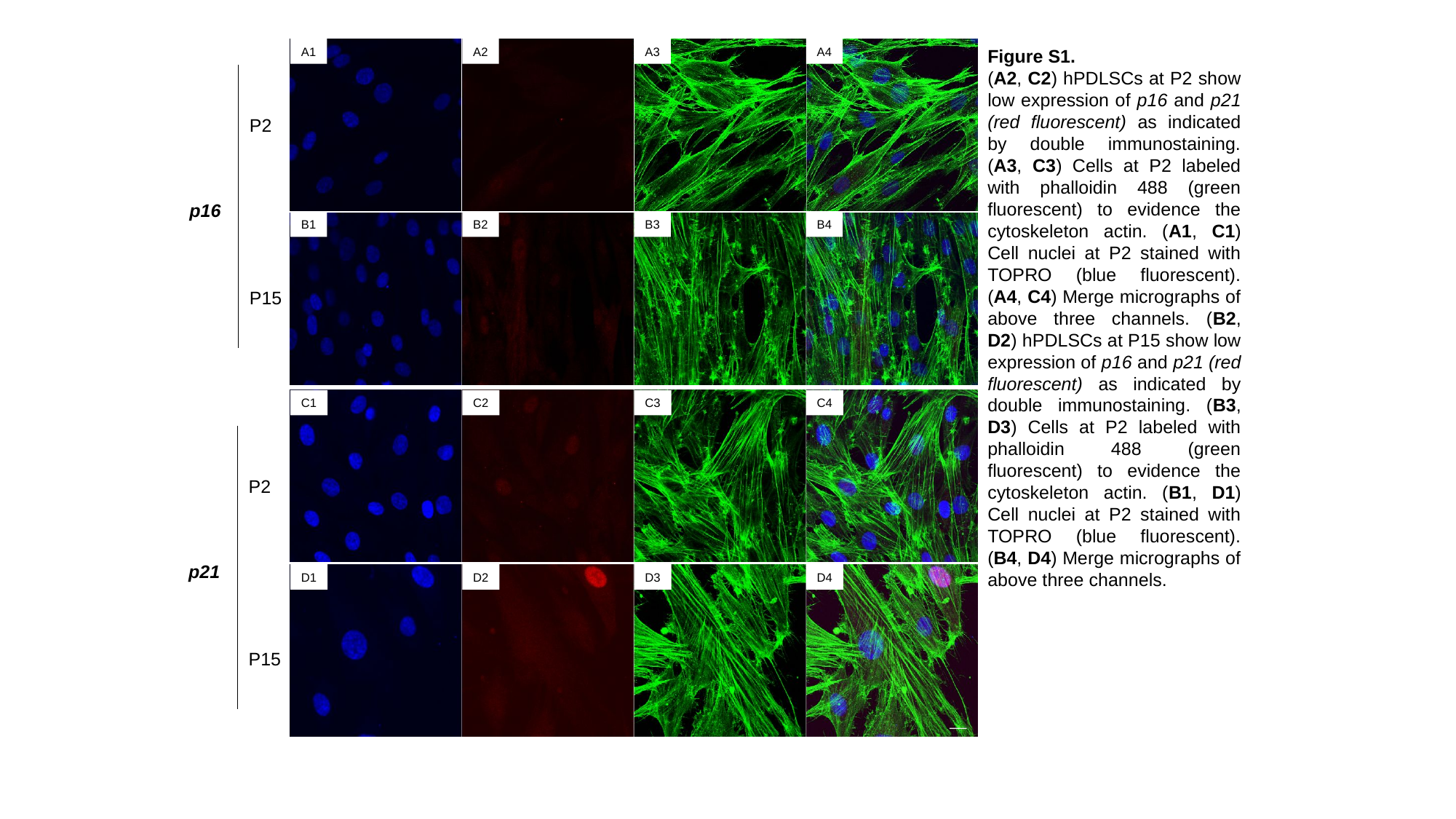

A1
A2
A3
A4
Figure S1.
(A2, C2) hPDLSCs at P2 show low expression of p16 and p21 (red fluorescent) as indicated by double immunostaining. (A3, C3) Cells at P2 labeled with phalloidin 488 (green fluorescent) to evidence the cytoskeleton actin. (A1, C1) Cell nuclei at P2 stained with TOPRO (blue fluorescent). (A4, C4) Merge micrographs of above three channels. (B2, D2) hPDLSCs at P15 show low expression of p16 and p21 (red fluorescent) as indicated by double immunostaining. (B3, D3) Cells at P2 labeled with phalloidin 488 (green fluorescent) to evidence the cytoskeleton actin. (B1, D1) Cell nuclei at P2 stained with TOPRO (blue fluorescent). (B4, D4) Merge micrographs of above three channels.
P2
p16
B1
B2
B3
B4
P15
C1
C2
C3
C4
P2
p21
__
D1
D2
D3
D4
P15
